# Health-related quality of life and health literacy in patients with systemic mastocytosis and mast cell activation syndrome

**DOI:** 10.1186/s13023-022-02439-x

**Published:** 2022-07-29

**Authors:** Tobias Jürgen Schmidt, Julia Sellin, Gerhard J. Molderings, Rupert Conrad, Martin Mücke

**Affiliations:** 1 Center for Rare Diseases Bonn (ZSEB), University Hospital Bonn, Bonn, Germany; 2 Institute for Digitalization and General Practice, University Hospital Aachen, Aachen, Germany; 3 Center for Rare Diseases Aachen (ZSEA), University Hospital Aachen, Aachen, Germany; 4 Institute of Human Genetics, University Hospital Bonn, Bonn, Germany; 5 Department of Psychosomatic Medicine and Psychotherapy, University Hospital Bonn, Bonn, Germany; 6 Department of Psychosomatic Medicine and Psychotherapy, Münster, Germany

**Keywords:** Systemic mastocytosis (ORPHA:2467), Mast cell activation syndrome, Health related quality of life, Health literacy

## Abstract

**Background:**

Systemic mastocytosis is a rare genetic disease characterized by aberrant proliferation and/or activation of mast cells, resulting in multi-organ, allergy-like symptoms. Mast cell activation syndrome (MCAS) is a clinically similar, but more prevalent disease with unclear etiology. In this study, the health-related quality of life (HRQOL) and health literacy of people suffering from SM and MCAS were assessed.

**Results:**

Two validated questionnaires (QLQ-C30/QLQ-INFO25) from the European Organisation for Research and Treatment of Cancer (EORTC) were used to analyze HRQOL and level of information of SM and MCAS patients. In addition, a control group without any health issues was included. Data were analyzed by ANOVA and linear regression to detect significant differences. Questionnaire data from 66 patients with MCAS (83% female, mean 44 years), 32 patients with SM (78% female, mean 53 years) and 52 healthy participants (67% female, mean 48 years) resident in Germany were analyzed. HRQOL as measured by the Global health status was significantly worse in patients suffering from MCAS or SM compared to control group. Individuals with MCAS showed a slightly, but insignificantly lower score on Global health status, and a significantly lower score with respect to role function and fatigue. Patients with the rare disease SM felt significantly better informed on their disease compared to MCAS patients. Linear regression performed separately for both groups showed a direct influence of the level of information on patients' HRQOL.

**Conclusion:**

Overall, our study showed a significant negative impact on the HRQOL of both diseases, but only a small difference in quality of life and severity of symptoms between patients with MCAS and the supposedly more severe form, the rare disease SM. Our results demonstrate that the level of information patients receive impacts HRQOL, and that this is not only an issue in rare diseases, but also diseases with unclear etiology and pathology. Our data shows that even slight improvements in the patient's level of information can have a positive effect on their quality of life, further highlighting the importance of gaining more knowledge on rare and incompletely understood diseases and communicating these insights to patients.

## Introduction

### Mast cell activation disease

The generic term mast cell activation disease (MCAD) encompasses a very heterogeneous group of primary mast cell diseases, including systemic mastocytosis (SM) and subtypes, the mast cell activation syndrome (MCAS), and mast cell leukemia (MCL) [1]. They are characterized by an aberrant release of mast cell mediators, mostly as the result of accumulation of either morphologically altered and immunohistochemically identifiable mutated mast cells from increased mast cell proliferation, or morphologically ordinary mast cells due to decreased apoptosis as well as an increased or prolonged state of activity [1]. As a result of inappropriate mediator release, allergy-reminiscent symptoms such as flushing, abdominal cramping, nausea, vomiting, pain as well as pruritus can occur [2].

### Systemic mastocytosis

Systemic mastocytosis is a rare disease with a prevalence of 0.3–13: 100.000 people in Europe [3]. As a proliferative disease of mast cells, it belongs to the primary mast cell activation diseases, which have in common that mutations in mast cells lead to abnormal proliferation, apoptosis, as well as increased and prolonged activation of the mutated mast cells [4].

Functionally activating somatic mutations of mast cell kinases and receptor proteins are thought to be the cause of systemic mastocytosis. Most frequently described is a point mutation in the gene 

coding for the KIT tyrosine receptor kinase CD117 (KIT^D816V^) [5]. Systemic mastocytosis presents predominantly in adults and is broadly classified into five main variants based on differences in histological findings and etiopathology [5–7]. Indolent SM (ISM) is the far most common form with a moderate mast cell accumulation in the bone marrow and possibly other organs [5–7]. The prognosed life expectancy of patients depends on the form of mastocytosis and can range from a normal life expectancy in the case of cutaneous SM (a form of mastocytosis that is limited to the skin) and ISM, to a few years or just months as with mast cell leukemia [4].

The clinical presentation of systemic mastocytosis can show a variety of different symptoms depending on the form and severity of the disease [4, 5]. In up to 80% of the cases, noticeable skin lesions occur in the form of brown–red maculopapular skin lesions 0.5 cm in diameter, which can be provoked by physical stimulus as a result of mast cell mediator release, resulting in local redness, urticarial swelling, and pruritus (also known as Darier's sign) [5]. Gastrointestinal symptoms in the form of colic-like abdominal pain, nausea, vomiting, and sudden attacks of diarrhea, tachycardia, hypotension, or flushing due to mediator release can also occur in all types of systemic mastocytosis, along with pronounced reactions to insect venoms, up to and including allergic shock, allergic reactions to drugs, gastrointestinal ulcers and secondary osteoporosis [4, 8].

In addition, other unspecific symptoms are often reported, like speech difficulties, musculoskeletal symptoms, fatigue, depression, unexplained weight loss, reflux, organ enlargement (like spleen and liver), anemia and eosinophilia [9].

For the diagnosis of systemic mastocytosis, the current WHO criteria apply for the fulfillment of which, among other tests, a bone marrow biopsy is essential. Further classification into subcategories of SM is based on additional histological criteria that will not be discussed in detail here [7].

### Mast cell activation syndrome

The mast cell activation syndrome (MCAS), as a variant of primary mast cell activation diseases, is a clinically extremely heterogeneous disease, which is still not fully understood with respect to its etiology and pathology, with the consequence of a more difficult diagnostic process [2].

According to current knowledge, mast cell activation syndrome is most likely the result of a variety of possible mutations, for instance mutations in kinases, receptors and proteins of signal transduction, inducing pathologically activated mast cells in different organ systems [10]. In contrast to systemic mastocytosis, an activating *KIT* point mutation in codon 816 is absent [2].

MCAS symptoms occur episodically with subsequent remission and, as the disease progresses, symptom-free intervals often become shorter [10]. Depending on the organ system affected, the symptoms can vary and resemble those of systemic mastocytosis [10]. The mast cell-mediated symptoms may include sudden onset of tachycardia, hypotensive syncope, dizziness, flushing, urticaria, angioedema, pruritus, abdominal cramps, nausea, vomiting, diarrhea, rhinorrhea, sneezing, wheezing, impaired concentration, fatigue as well as inflammation of the mucosa of the gastrointestinal tract and the respiratory tract [10, 11]. The combination of several of these listed symptoms occurring in different organ systems indicates the presence of MCAS [11].

Due to the extremely heterogeneous symptomatology, depending on which organ system is affected, the diagnosis of MCAS proves rather difficult. Up to now, it has to be made primarily based on clinical findings and diagnostic criteria consisting of some laboratory parameters and immunohistochemical findings in biopsies. Currently, two approaches to diagnose MCAS are discussed, termed Consensus-1- [12] and Consensus-2-criteria [13] (containing some differences in the interpretation of the criteria) suggested by two different expert groups. Following the Consensus-2 criteria, up to 17% of the German population are suspected to suffer from MCAS [8]. It is therefore by no means a rare disease, but due to the lack of knowledge about etiology and pathology, in combination with often unspecific clinical presentation, MCAS patients suffer from delayed diagnosis and misdiagnoses, often for decades. Further delay in access to effective, quality-of-life-improving treatment should therefore be prevented at all cost.

### SM and MCAS and health-related quality of life

Assessing the Health-related quality of life (HRQOL) of patients suffering from SM and MCAS is important for the evaluation of therapy, but also to increase the awareness of the impact of these different variants of MCAD on daily life and well-being. So far, there are published data only on the HRQOL of patients suffering from SM, showing convincingly that SM does have a negative impact on the HRQOL of affected individuals [14–17]. Given that MCAS has similar symptoms as systemic mastocytosis, we hypothesized that MCAS also has a negative impact on health-related quality of life just as systemic mastocytosis. Due to the still very inconsistent definition of MCAS, the numerous suspected triggering or exacerbating mutations (whose exact influence on the clinical pattern is still unclear), potential environmental or individual factors, and the diverse clinical course of the disease, we assumed that MCAS is more variable in its presentation. With the significantly reduced HRQOL of patients with SM already demonstrated in other studies in mind, we therefore hypothesized that at least some of the MCAS patients show a milder influence on QoL in direct comparison with SM [14–17]. Therefore, the aim of this study was to compare the health-related quality of life and the specific symptom burden of both chronic mast cell diseases to one another as well as to healthy controls. In this context it was of particular interest to investigate whether fatigue, which is known to have a major impact on quality of life and activities of daily living in a vast spectrum of chronic diseases such as cancer, end-stage renal, liver, or lung diseases, as well as CNS pathologies and many others, would also be a more consistent factor in SM compared to MCAS [18–22].

As there is a lot of uncertainty and lack of information particularly regarding MCAS, even among physicians, we were interested in the impact of patients’ level of information on HRQOL. Therefore we wanted to find out whether there is a difference between patients with systemic mastocytosis and patients with mast cell activation syndrome with regards to the level of information they have about their respective disease, with a focus on the information supplied by the treating physicians. Additionally, we wanted to analyze whether a higher level of information on these two mast cell disease variants is associated with a higher health-related quality of life, a fact that has already been proven to be true for patients with other chronic diseases [23–25].

Taken together, we formulated the following hypotheses:

1. SM and MCAS show a lower HRQOL compared to the healthy control group as measured with the Global symptom burden of the QLQ-C30.
2. There is lower HRQOL in SM compared to MCAS as measured with Global symptom burden of the QLQ-C30.
3. Regarding specific symptom burden, particularly fatigue as a major debilitating symptom is significantly higher in SM and MCAS compared to healthy controls.
4. Fatigue as a particular debilitating symptom is higher in SM compared to MCAS.
5. MCAS patients show a significantly lower MCAS-related global health literacy score compared to SM as measured by the INFO-25
6. The degree of disease-related health literacy as measured by the INFO-25 Global Score significantly predicts HRQOL in SM as well as MCAS in linear regression.

## Results

A total of 98 patients from Germany participated in the study, including 66 with mast cell activation syndrome and 32 patients with systemic mastocytosis (see Table 1). In addition, there was a control group consisting of 52 participants without any health issues (see Table 1). No incomplete or invalid questionnaires were provided by the participants; therefore the results of no participant had to be excluded from this study.

**Table 1:** Group characteristics

	MCAS	SM	Control group
Number of patients	N = 66	N = 32	N = 52
Sex			
Female	55 (83%)	25 (78%)	35 (67%)
Male	11 (17%)	7 (22%)	17 (33%)
Age			
Mean	44.18	53.12	47.82
Range	18–77	32–76	22–78

The χ^2^ test did not reveal any significant differences with regard to gender (*p* 0.169) and age (*p* 0.171) between the three groups.

### HRQOL differences between MCAS, SM, and control group

**Fig. 1:**
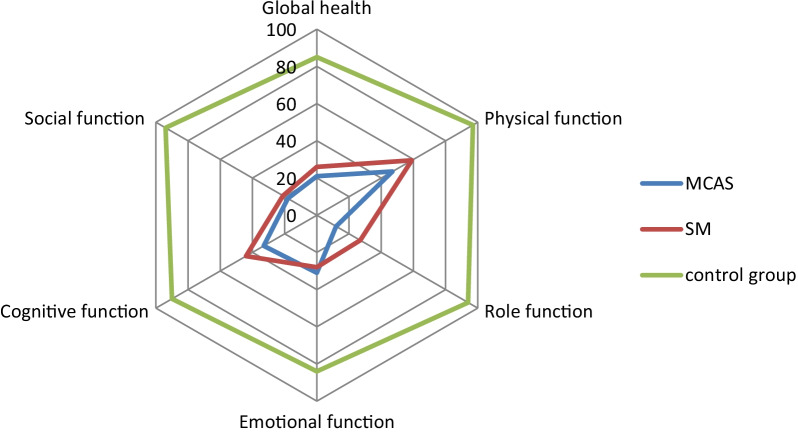
Mean values of the individuals’ functional scales and global health score (assessed with the EORTC QLQ-C30) of the MCAS, SM and Control Group. Functional scales on a scale from 0 to 100, with higher scores indicating a better the Health-Related Quality Of Life of the groups’ participants in total

Comparing the global health status of the three groups revealed a clear difference between the two mast cell diseases and the healthy control group confirming our first hypothesis. Furthermore, the comparison between each of the disease groups and the control group showed for all five functional scales significant differences in the Games-Howell post hoc test. (see Table 2 and Fig. 1). The effect sizes as measured by Cohen's d when comparing the two mast cell diseases and the control group were markedly above 0.8, ranging from 2.21 to 3.89, corresponding to a very strong effect.

**Table 2:** EORTC-QLQ-C30 scores

Variable	MCAS	SM	Control group	Welch´s—F	MCAS vs. SM	MCAS vs. C	SM vs. C
	N = 66	N = 32	N = 52		*p* Value*/Cohens´s d	*p* Value*/Cohen´s d	*p* Value*/Cohen´s d
Global health status	21 ± 17	26 ± 19	85 ± 12	330.72*	0.413	**< 0.001/4.32**	**< 0.001/3.89**
Functioning scales							
Physical function	47 ± 24	59 ± 26	97 ± 6	156.37*	0.079	**< 0.001/2.72**	**< 0.001/2.26**
Role function	12 ± 20	27 ± 30	94 ± 14	353.97*	**0.038/0.62**	**< 0.001/4.64**	**< 0.001/3.15**
Emotional function	31 ± 24	28 ± 22	84 ± 18	116.90*	0.762	**< 0.001/2.39**	**< 0.001/2.83**
Cognitive function	33 ± 30	44 ± 27	90 ± 16	105.07*	0.192	**< 0.001/2.30**	**< 0.001/2.21**
Social function	18 ± 22	21 ± 28	94 ± 17	249.82*	0.844	**< 0.001/3.85**	**< 0.001/3.31**

Significant *p* values and corresponding Cohen's d are marked in bold type

Welch-ANOVA with *Games–Howell post-hoc test for calculation of *p* values between MCAS and SM, MCAS and control group, SM and control group; mean value ± standard deviation of the three groups; Cohen´s d as effect size; *Welch’s F *p* < 0.001; C = Control Group

**Fig. 2:**
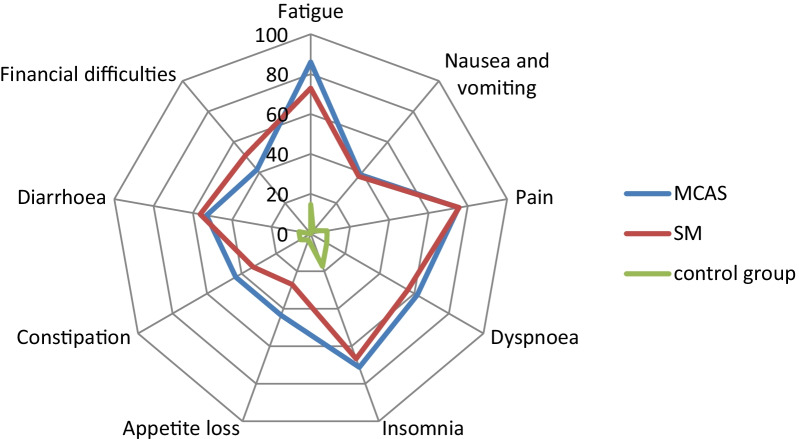
Spider plot showing EORTC QLQ-C30 symptom scores of MCAS, SM and Control Group. Symptom scores on a scale from 0 to 100, with higher scores indicating a higher number of medical conditions reported

Directly comparing the results of the MCAS patient group with the SM patient group on global health status, there was no significant difference that could have confirmed our second hypothesis. As a matter of fact, the overall influence of SM on the individual scales and symptoms showed a non-significant tendency towards a slightly less pronounced impact than for MCAS disease (see Figs. 1, 2—Spider plot). A statistically significant difference in the scales is only found in the Role function in the Games-Howell post-hoc test (*p* < 0.038) with a Cohan's d of 0.62, corresponding to a moderate effect size, indicating that MCAS patients report a higher impact of their disease on role function than SM patients. Both groups achieved their respectively highest values for physical function, as for instance with physical resilience (see Fig. 1—Spider plot).

### Symptom burden differences between MCAS, SM and control group

Comparing the results of the two patient groups with those of the control group consisting of healthy individuals, there were significant differences in all symptom scores (*p* < 0.001), confirming our third hypothesis on fatigue. Again, the Cohen's d effect size is shown to be significantly greater than 0.8 when comparing the two mast cell diseases and the control group in each case, indicating once again a strong effect (see Table 3). This and the results of the functional scales further demonstrate the pronounced impairment on health-related quality of life of patients with mast cell disease.

**Table 3:** EORTC-QLQ-C30 symptom scales/items

Symptom burden	MCAS	SM	Control Group	Welch´s-F	MCAS vs. SM	MCAS vs. C	SM vs. C
	N = 66	N = 32	N = 52		*p* Value*/Cohens´s d	*p* Value*/Cohen´s d	*p* Value*/Cohen´s d
Fatigue	86 ± 20	73 ± 26	15 ± 19	193.60**	**0.041/0.59**	** < 0.001/3.60**	** < 0.001/2.61**
Nausea and vomiting	39 ± 25	38 ± 28	2 ± 9	75.92**	0.979	** < 0.001/1.83**	** < 0.001/1.91**
Pain	75 ± 30	76 ± 28	9 ± 15	168.56**	0.999	** < 0.001/2.73**	** < 0.001/3.19**
Dyspnoea	62 ± 32	56 ± 36	10 ± 19	70.27**	0.758	** < 0.001/1.93**	** < 0.001/1.75**
Insomnia	71 ± 32	67 ± 31	17 ± 21	71.98**	0.776	** < 0.001/1.95**	** < 0.001/1.97**
Appetite loss	43 ± 37	27 ± 32	3 ± 10	40.46**	0.072	** < 0.001/1.40**	** < 0.001/1.12**
Constipation	43 ± 37	33 ± 35	6 ± 19	27.75**	0.394	** < 0.001/1.22**	** < 0.001/1.04**
Diarrhoea	53 ± 34	56 ± 33	6 ± 19	60.24**	0.896	** < 0.001/1.64**	** < 0.001/1.97**
Financal difficulties	42 ± 42	51 ± 41	0 ± 0	–***	0.568	** < 0.001/1.34**	** < 0.001/2.00**

Significant *p* values and corresponding Cohen's d are marked in bold type

Welch-ANOVA with *Games-Howell post-hoc test for calculation of *p* values between MCAS and SM, MCAS and Control Group, SM and control group; mean value ± standard deviation of the three groups; Cohen´s d as effect size; **Welch’s F *p* < 0.001; *** = cannot be calculated, because in at least one group a variance of 0 occurs; C = Control Group

The medical conditions reported by the MCAS patients as well as by the SM patients show a similar distribution: Fatigue, pain, and insomnia were by far the most frequently reported complaints, followed by dyspnea and diarrhea as other main complaints (see Table 3 and Fig. 2—Spider Plot). Interestingly, there is a non-significant tendency for individuals affected by MCAS to suffer slightly more of symptoms like constipation and appetite loss and from financial difficulties due to their disease than those with SM. (see Fig. 2—Spider Plot). Yet, a significant difference between the two groups was seen in the Games-Howell post hoc test only for the symptom of fatigue (*p* < 0.041), which was reported to be more pronounced in MCAS than in SM. With a Cohen's d of 0.59, this corresponds to a moderate effect size (see Table 3). This is in stark contrast to our fourth hypothesis, which assumed a significantly higher fatigue score in SM patients.

### Comparing the health literacy of MCAS and SM patients using the EORTC QLQ-INFO25

First of all, we wanted to determine if there is a significant difference in the health literacy of the patients of these two diseases with regards to the information they have received by their attending physicians. The results show that, in general, the patients of both groups do not feel very well informed by the treating physicians about their disease. Almost all patients from both mast cell diseases (99% of the cases) would like to receive more information about their medical condition, which is reflected in the RECMORE scale (patient would like to receive more information). Accordingly, only one patient (in this case a patient affected by SM) wanted less information as can be seen from the results of the RECLESS (patient would like to receive less information) scale. In response to an open question, most frequently, the patients of both groups expressed a desire for more information about the causes, possible familial inheritance, as well as current and future treatment options for these diseases.

In the Welch test, significant differences between the level of information of patients with mast cell activation syndrome and those with systemic mastocytosis are noticeable. The global score differed significantly with a medium to strong effect size confirming our fifth hypothesis (level of information in MCAS worse than in SM). Looking at the different subscales INFODIS (information about the disease itself, its cause and extent as well as the current disease control), INFOMEDT (information about the purpose, the procedures and the results of performed diagnostics), INFOTREAT (information about the different treatment options, their advantages and expected impact on the symptoms of the disease as well as possible side effects of these treatments), INFOHELP (information about ways to positively improve the course of the disease), SATINFO (satisfaction with the amount of information received) and OVERHELP (information overall was considered helpful by the patient) show significant differences (see Table 4). In summary, patients with systemic mastocytosis were significantly more satisfied with the amount and quality of information received from their treating physicians in the final global score calculated from all scales (*p* < 0.001—see Table 4). The effect size comparing the two mast cell disease variants, represented by Cohen's d, is above 0.8 for the variables INFODIS and INFOTREAT, which corresponds to a strong effect. For the variables INFOMEDT, SATINFO as well as OVERHELP, a moderate effect is shown. The effect for the variable INFOHELP is rather small (see Table 4).

**Table 4:** EORTC-QLQ-INFO25 scores

Variable	MCAS	SM	T	df	*p* value	Cohen´s d
	N = 66	N = 32				
Global score	23 ± 13	33 ± 13	− 3.50	58.01	**< 0.001**	**0.77**
INFODIS	19 ± 18	35 ± 23	− 3.49	50.53	**< 0.001**	**0.81**
INFOMEDT	34 ± 28	52 ± 22	− 3.47	75.15	**< 0.001**	**0.69**
INFOTREAT	15 ± 14	28 ± 18	− 3.66	50.53	**< 0.001**	**0.85**
INFOTHSE	13 ± 17	21 ± 21	− 1.76	51.33	0.085	0.41
INFODIFP	3 ± 9	6 ± 16	− 1.25	40.91	0.219	0.32
INFOHELP	21 ± 24	32 ± 26	− 2.12	56.98	**< 0.05**	**0.47**
INFOWRIN	32 ± 47	53 ± 51	− 2.00	57.39	0.05	0.44
INFOCD	0	0	–	–	–	–
SATINFO	17 ± 22	30 ± 27	− 2.45	51.38	**< 0.018**	**0.57**
RECMORE	100 ± 0	97 ± 17	− 1.00	31.00	0.325	0.31
RECLESS	0	3 ± 17	1.00	31.00	0.325	0.31
OVERHELP	27 ± 28	42 ± 24	− 2.75	69.86	**< 0.01**	**0.56**

Significant *p* values and corresponding Cohen's d are marked in bold type

Welch test for calculating the *p* values of the individual scales of the QLQ-INFO25 for MCAS and SM, means ± standard deviation; Cohen´s d as effect size; INFODIS, information about disease; INFOMEDT, about medical tests; INFOTREAT, about treatments; INFOTHSE, about other service; INFODIFP, different places of care; INFOHELP, things you can do to help yourself; INFOWRIN, written Information; INFOCD, digital information; SATINFO, satisfaction with the information received; RECMORE/RECLESS, wish to receive more/less information; OVERHELP, overall the information has been helpful

No significant differences between both groups were found in the following scales: INFOTHSE (information about other services to manage the disease), INFODIFP (information on different places of care), RECLESS, and RECMORE (see Table 4). With a *p* Value of 0.05, the variable INFOWRIN (written information provided) also shows no significant difference, albeit borderline. INFOCD (digital information provided) cannot be calculated because the standard deviations of both groups are equal to 0.

**Fig. 3:**
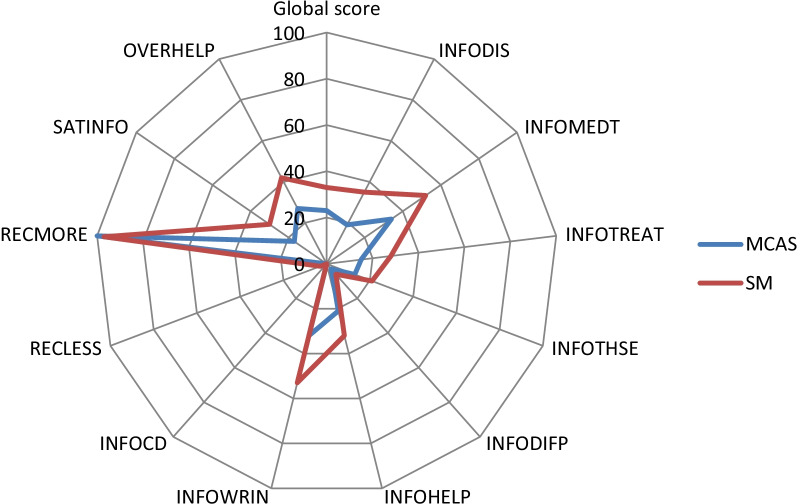
Mean values of the individual scales of the EORTC QLQ-INFO25 depending on the group membership MCAS/SM; Level of information scores on a scale from 0 to 100, with higher scores indicating a better level of information; INFODIS, Information about disease; INFOMEDT, about medical tests; INFOTREAT, about treatments; INFOTHSE, about other service; INFODIFP, different places of care; INFOHELP, things you can do to help yourself; INFOWRIN, written Information; INFOCD, digital information; SATINFO, satisfaction with the information received; RECMORE/RECLESS, wish to receive more/less information; OVERHELP, overall the information has been helpful; the higher the score, the better the information level

The highest satisfaction scores in the two groups present in the subscales INFOMEDT, INFOWRIN and OVERHELP (see Table 4 and Fig. 3). At least as far as the diagnostics performed on them and their results are concerned, many of the patients felt informed to some extent. They also felt that the little information they were given was helpful. None of the participating patients received information in digital form (INFOCD) from their treating physicians.

To show the influence of health literacy—achieved by the treating physicians—of a patient with mast cell disease on their perceived health-related quality of life, we performed a linear regression for both patient groups with the global quality of life score collected in the QLQ-C30 as the dependent variable and the Global Score, calculated by the individual subscales of the QLQ-INFO-25 as the independent variable. As previously hypothesized (hypothesis 6), we expected a correlation between health literacy and quality of life, which was especially interesting in the light of better performance of the SM group on the QLQ-INFO25 and the overall slightly better performance on the QLQ-C30, as an effect caused by health literacy could be a possible explanation.

In the linear regression model with the factor Global Score, a goodness of 0.75 (corrected R^2^) for MCAS and 0.59 for SM was achieved, which means 75%/59% of the variance of quality of life were explained by this predictor (see Table 5) and corresponds to a very good model. The Global Score proved to be similarly strong in both groups (standardized regression coefficients beta MCAS = 0.866 and SM = 0.775).

**Table 5:** Univariate linear regression models for MCAS and SM

	Non-standardized coefficients		Standardized coefficients	T	Sig.*	Corrected R^2^
	Regression coefficient B	Std. failure	Beta			
Global Score (MCAS)	1.143	0.082	0.866	13.878	< 0.001	0.75*
Global Score (SM)	1.107	0.165	0.775	6.719	< 0.001	0.59*

Linear regression model with Global health status as the dependent variable; Global Score = calculated by the individual INFO25-subscales

The non-standardized regression coefficients B show the change of the dependent variable (in our case the global health status) with one step of change of the independent variable (in our case the Global Score of the QLQ-INFO25). For example, if Global Score in the MCAS group increases by one scale point, Global health status increases by 1.143 scale points (for SM group see Table 5).

In conclusion, by combining both questionnaires and comparing the results, we were able to confirm that a better health literacy, through information, of people suffering from MCAS or SM has a positive effect on their perceived health-related quality of life.

## Discussion

To this date, there are only a few published data on the health-related quality of life of individuals suffering from systemic mastocytosis, all of which impressively show its negative impact [14–17]. However, no data are available on this subject for mast cell activation syndrome. With this in mind, our study investigated respective mast cell diseases and analyzed their impact on individual well-being.

In our study the overall gender distribution in mast cell activation syndrome shows significantly more female than male participants (see Table 1), which is consistent with the prevalence distribution previously found in studies [26–28]. However, as systemic mastocytosis usually does not show such a pronounced skewed distribution (see comparative studies [29, 30]), one should also take the possibility into consideration that female patients afflicted by a disease (be it SM or MCAS) possibly show a greater interest in their disease and also in actively participating in a research study, which probably also influenced the gender distribution of this study’s participants. In terms of age distribution, the MCAS group showed rather an even distribution from young to old. The group of SM included fewer young patients. This may be due to the fact that SM during childhood and adolescence is frequently limited to the skin and can hardly be distinguished from cutaneous mastocytosis, and those patients were not included in our study [31]. The adult form usually manifests between the ages of 20 and 40, and a final diagnosis is often not made until after the age of 30 [32].

We were able to reveal large differences in health-related quality of life not only between patients with SM and a healthy control group—as has been demonstrated in other studies—but also between patients with MCAS and healthy individuals, confirming our first hypothesis. The global quality of life status, all functional scales, symptom scales, and individual symptoms were significantly decreased. The very large effect sizes highlight the high degree of impairment and suffering of affected patients.

In contrast to our second hypothesis, there was no significant difference between both diseases regarding the global health status. MCAS patients even had a slightly, although non-significant lower HRQOL on several subscales, and the subscale role functioning was even significantly lower. This degree of functional impairment underlines the necessity of improved diagnostics and therapy for this disease. It could be speculated that the cause of the differences in health-related quality of life between MCAS and SM patients would fit to an epigenetic/genetic distortion of the function of the affected mast cells, which might warrant further research in the future.

As assumed in our third hypothesis, there is a significant influence of the two mast cell diseases on fatigue as one of the main symptoms compared to the healthy control group. In systemic mastocytosis and MCAS, both classified as hematologic neoplasms, fatigue, pain and insomnia are among the most frequent or predominant complaints, similar to a variety of different cancers such as ovarian cancer, epithelioid hemangioendothelioma or neuroendocrine tumors [33–35].

In our fourth hypothesis we assumed that fatigue as a particular debilitating symptom is significantly higher in SM compared to MCAS. However, the comparison of the two mast cell diseases showed significant differences in the psychosocial domain in the form of fatigue and limitations in daily life and work (role function), with a more prominent expression in the MCAS group. This result was unexpected for us given our initial assumptions and thus our fourth hypothesis was rejected. One possible reason for this result could be the often very long and psychologically very stressful diagnostic procedure MCAS patients have to go through due to the unclear etiology and lack of general knowledge about their disease. Similar to what is often found with rare diseases, like SM, MCAS patients also receive the correct diagnosis often only after decades, and many MCAS patients have had countless visits to various doctors and hospitalizations with the same examinations over and over again [26]. A major difference to SM is however the clarity of diagnostic tests, as SM can be confirmed genetically or histologically, while the diagnosis of MCAS is still a topic of debate among researchers [2, 7]. Furthermore, the severity of fatigue could also be the cause of the significant difference in daily life and work (role function) between the two diseases, a connection which has already been discussed in other publications [22, 36].

In our fifth hypothesis we assumed that MCAS patients show a significantly lower disease-related health literacy score compared to SM as measured by the INFO-25. In keeping with our hypothesis, we were able to detect a significant difference between patients with MCAS and SM in the subjective level of information they have about their disease. Patients with SM were significantly more satisfied by the information they have received from their attending physicians confirming our fifth hypothesis. This is an interesting aspect of the two studied related diseases, as the rare disease among them is better characterized than the more prevalent one. Despite its low prevalence, SM, first described in the middle of the twentieth century, is better known and understood by the treating physicians (including its genetic etiology), and, hence, can be better explained to the patients. In addition, there are established self-help groups, which provide additional information. On the other hand, the mast cell activation syndrome, which for a long time was not recognized as a systemic mast cell disease, is in the focus of scientific research for only about two decades and is not as present in physicians’ minds as SM [2]. The current situation with two diagnostic criteria for MCAS may add to the uncertainty [2, 12]. However, while the two current consensus criteria (consensus criteria 1 [12] vs. 2 [2]) differ slightly with regard to the number of cases that fall under their cut-off, they are an important step towards raising increasing awareness in the medical community and will hopefully find wider use in the future, with the potential to shorten time to diagnosis. We are optimistic that an overarching compromise will be reached soon to reconcile the differences.

As formulated in our sixth hypothesis, the degree of disease-related health literacy significantly predicted HRQOL in SM as well as MCAS in linear regression. The level of information about the disease has a significant positive impact on the health-related quality of life of patients with MCAS as well as SM. This, in combination with the significantly higher level of information of the SM group, could at least partly explain the better results of the SM group in the first part of our study. When the treating physician provides well-chosen, specific information and details to the patient, even if it is only an explanation of the planned diagnostic steps, their benefits, and their results (regardless of the suspected underlying disease), an increase in the patient's perceived health-related quality of life can occur. Of course, this is only possible if the attending physician is sufficiently well informed about the disease. For very rare diseases such as systemic mastocytosis, which a physician probably runs across only in special medical centers, further improvement in the information level of physicians might be hard to achieve. However, as for mast cell activation syndrome with an assumed prevalence of up to 17% in the German population and which occurs therefore quite frequently, physicians should be well advised to get familiarized with this disease in order to be able to detect it fast, communicate it well to the patient, and thereby help them to get the right treatment as soon as possible [3]. Several other studies, for instance in patients with cancer, have already demonstrated a positive impact of health literacy on quality of life in varying but significant degrees [23–25]. A well-informed patient who is satisfied with the information provided by the treating physician is likely to cope better with the illness in everyday life than someone who is burdened by dissatisfaction due to a lack of disease-related information in addition to the symptomatology. It may therefore also be easier for these well-informed patients to take part in further diagnostic steps and also be more willing to try further therapy options or to accept other help options that could make life easier.

## Conclusions

In conclusion, this study has shown that both, SM as well as MCAS have a significant negative impact on the quality of life of affected individuals. Overall, MCAS patients seem to be more affected, particularly in the psychological domain as well as in everyday and work life. Patients with SM appear to have received significantly more information from their treating physicians about their disease, which is reflected in an increased level of satisfaction and which appears to have a positive impact on the health-related quality of life.

## Methods and material

### Setting and population

In this study, patients with confirmed MCAS or SM who are resident in Germany and more than 18 years of age were included. Affected individuals for the systemic mastocytosis group were recruited after diagnosis according to criteria specified by WHO [7]. For the MCAS group, the Consensus-2 criteria (Afrin et al. 2020) including histological and laboratory diagnostic criteria for pathological mast cell activity were used [2]. All patients were recruited after having been diagnosed and before initiation of therapy or without ongoing therapy. Participants for a healthy control group, were locally recruited, aiming for a similar age and gender distribution as in the SM and MCAS groups. An age above 18 years and a healthy general condition at the time of the interview were defined as inclusion criteria. Chronic diseases were considered exclusion criteria.

The study design was reviewed and approved by the local ethics committee, registered through the German Register of Clinical Studies (Deutsches Register klinischer Studien, DRKS) with the study ID DRKS00015691 on the 2nd of September 2019 and performed in accordance with the Declaration of Helsinki and in compliance with Good Clinical Practice. Written informed consent was obtained from each patient.

### Questionnaires used to collect data

Validated questionnaires (QLQ-C30 and QLQ-INFO25) from the European Organization for Research and Treatment (EORTC) were used, which have already been utilized in various other studies in the past and proved effective. The questionnaires were originally developed to assess the health-related quality of life as well as health literacy of patients with malignant diseases and can thus also be used for systemic mastocytosis and MCAS, as both variants are categorized as hematologic neoplasia due to multiple epigenetic/genetic alterations.

The European Organization for Research and Treatment (EORTC) is an international, non-profit organization based in Brussels that conducts studies on cancer therapies with a focus on increasing the survival rate as well as health related quality of life of cancer patients. Among others, they have developed the QLQ-C30 (version 3.0), a questionnaire with a total of 30 items to assess the health-related quality of life [37]. The first 28 questions are answered on a four-point Likert scale (1—not at all, 2—a little, 3—moderately, 4—very much) and finally the last two questions on a seven-point scale (1—very poor to 7—excellent) for general health and quality of life. In the evaluation, these questionnaire items contribute data to 5 functional scales (physical, role, cognitive, emotional and social scale), a global quality of life scale, 3 symptom scales as well as individual items on specific symptoms and the financial burden the disease has on patients afflicted by it. Subsequently, the individuals’ answers are converted into scales with values from 0 to 100 according to the EORTC scoring manual. The higher the value of the functional scales and the global quality of life scale, the better the physical/psychological function and the overall quality of life of the patient. The symptom scales, on contrast, have exactly the opposite effect. For reliability analysis, Cronbach’s alpha was calculated to assess the internal consistency of the subscales. The internal consistency of the questionnaire is satisfying, with a Cronbach’s alpha for the functional scales, the symptom scales and the global health status with a range from 0.89 to 0.97.

The QLQ-INFO25-questionnaire, also developed by the EORTC, is a supplementary module to the QLQ-C30-questionnaire and consists of 25 additional questions, which deal with the information the patient has received previously by the treating physicians, for example about the disease itself, the diagnostics performed as well as about the diagnostic results and the ensuing therapy [38]. It is divided into 21 questions with a four-point Likert scale (1—not at all, 2—a little, 3—moderately, 4—very much) and 4 questions offering a YES/NO answer option with regard to the receipt of written or digital information and the desire for more or less information, including the option of specifying certain desired information. The evaluation, based on the scoring manual, divides the questionnaire into 12 scales and a global score with values from 0 to 100. The twelve scales are divided into INFODIS (information about the disease itself, its cause and extent as well as the current disease control), INFOMEDT (about the purpose, the procedures and the results of performed diagnostics), INFOTREAT (about the different treatment options, their advantages and expected impact on the symptoms of the disease as well as possible side effects of these treatments), INFOTHSE (about other services), INFODIFP (about different places of care), INFOHELP (about ways to positively improve the course of the disease), INFOWRIN/-CD (received written/digital information), RECMORE/RECLESS (patient would like to receive more/less information), SATINFO (satisfaction with the amount of information received) and OVERHELP (information overall was considered helpful by the patient). The measured subscales can then be used to calculate the GLOBAL SCORE as the final assessment of the level of information. The higher the value, the better the patient's subjective level of information. The internal consistency of the questionnaire, shown by Cronbach's Alpha, is satisfying with a range from 0.73 to 0.92.

The QLQ-C30 was used in all three groups of this study (both patient groups as well as the control group) in order to assess the health-related quality of life, whilst the QLQ-INFO25 was only provided to the two groups of patients to compare their health literacy with focus on the level of information, provided by the attending physician, patients have about their disease and its diagnosis as well as treatment.

### Statistical analysis

SPSS Statistics, version 26.0 for Windows from IBM SPSS, Chicago, IL, USA, was used for the statistical analysis of this study. First, the individual calculated scales and items of the EORTC QLQ-C30 questionnaire of the three groups (MCAS patient group/SM patient group/Control Group) were compared with each other by means of Welch-ANOVA and Games-Howell as a post-hoc test, with the aim of checking for any significant differences between the three groups.

A Welch test was performed to check for any significant differences between the two patient groups as to how satisfied the patients are with regards to the information they have received from the treating physicians. Linear regression was performed to show the influence of individual items/scales of the EORTC QLQ-INFO-25 questionnaire on the global health status score, which was measured with the EORTC QLQ-C30 questionnaire.

## Data Availability

The datasets analyzed and discussed are available from the corresponding author upon request.
