# Correction : Health-related quality of life and health literacy in patients with systemic mastocytosis and mast cell activation syndrome

**DOI:** 10.1186/s13023-023-02645-1

**Published:** 2023-02-16

**Authors:** Tobias Jürgen Schmidt, Julia Sellin, Gerhard J. Molderings, Rupert Conrad, Martin Mücke

**Affiliations:** 1 Center for Rare Diseases Bonn (ZSEB), University Hospital Bonn, Bonn, Germany; 2 Institute for Digitalization and General Practice, University Hospital Aachen, Aachen, Germany; 3 Center for Rare Diseases Aachen (ZSEA), University Hospital Aachen, Aachen, Germany; 4 Institute of Human Genetics, University Hospital Bonn, Bonn, Germany; 5 Department of Psychosomatic Medicine and Psychotherapy, University Hospital Bonn, Bonn, Germany; 6 Department of Psychosomatic Medicine and Psychotherapy, University Hospital Münster, Münster, Germany

**Correction :  Orphanet Journal of Rare Diseases (2022) 17:295** 10.1186/s13023-022-02439-x

Following publication of the original article [1], we have been notified that affiliation 6 was incorrectly mentioned in the published article. It should be as follows:

Department of Psychosomatic Medicine and Psychotherapy, University Hospital Münster, Münster, Germany.
